# Myocardial tissue changes detected by cardiac MRI in a patient with suspected systemic sarcoidosis

**DOI:** 10.1186/s12872-023-03133-x

**Published:** 2023-03-11

**Authors:** Byambasuren Vanchin, Mame Madjiguène Ka, Christophe T. Arendt, Felicitas Escher, Eike Nagel, Valentina O. Puntmann

**Affiliations:** 1 Institute for Experimental and Translational Cardiovascular Imaging, DZHK Centre for Cardiovascular Imaging, University Hospital Frankfurt, Frankfurt Am Main, Germany; 2 Department of Cardiology, School of Medicine, Mongolia-Japan Teaching Hospital, Mongolian National University of Medical Sciences, Ulaanbaatar, Mongolia; 3 Department of Cardiology, University Hospital Berlin Charite, Berlin, Germany

**Keywords:** Systemic sarcoidosis, Myocardial inflammation, Cardiac remodeling, Tissue mapping, Scar

## Abstract

**Background:**

The role of cardiac magnetic resonance imaging in the early management of chronic cardiac inflammatory conditions is growing. Our case enlightens the benefit of quantitative mapping in the monitoring and treatment guidance in systemic sarcoidosis.

**Case presentation:**

We report about a 29-year-old man with an ongoing dyspnea and bihilar lymphadenopathy, suggesting sarcoidosis. Cardiac magnetic resonance showed high mapping values, but no scarring. In follow-ups, cardiac remodeling was noted; cardioprotective treatment normalized cardiac function and mapping markers. Definitive diagnosis was achieved in extracardiac lymphatic tissue during a relapse.

**Conclusion:**

This case shows the role that mapping markers can play in the detection and treatment at early stage of systemic sarcoidosis.

## Background

Cardiac involvement is the leading cause of death in systemic sarcoidosis [1], due to fatal arrhythmias, conduction abnormalities or heart failure. The Heart Rhythm Society (HRS) [2] and the Japanese Society of Sarcoidosis and other Granulomatous Disorders [3] has both required the presence of late gadolinium enhancement (LGE) at CMR for the clinical diagnosis of cardiac sarcoidosis, among other criteria using different cardiac imaging tools (echocardiography, radionuclide scintigraphy, or cardiac catheterization). However, these techniques are mainly based on the detection of advanced structural and/or functional damages of heart muscle. Quantitative tissue characterization which is related to altered magnetization of myocardial properties is found to be more sensitive method [4] and therefore, can allow an early recognition and treatment of cardiac sarcoidosis, before the detection of LGE. This case illustrates the role of quantitative tissue mapping in the early stages of the disease.

## Patient information

A 29-year-old previously healthy man presented for an outpatient respiratory assessment of shortness of breath and vertigo lasting for a year. Both symptoms were limiting his professional performance, demanding physical fitness and a high degree of sensorimotor acuity. There were no chronic communicable and non-communicable diseases of note. A year prior, he had a bronchopulmonary infection with protracted symptoms but complete resolution.

## Clinical findings

Clinical examination was normal.

## Timeline11


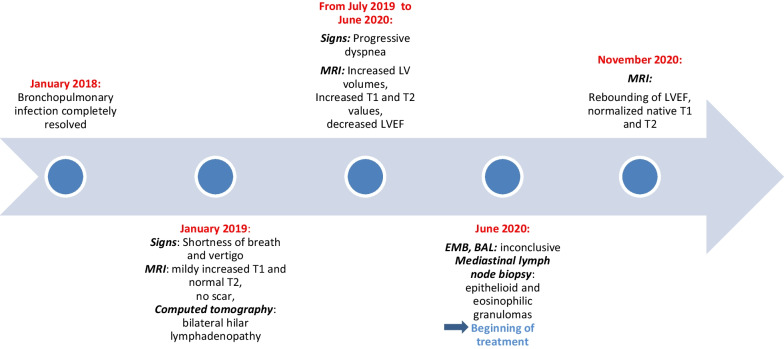


## Diagnostic assessment

The remarkable finding on initial investigations was bilateral hilar lymphadenopathy (BHL) at CMR, raising possibility of systemic sarcoidosis or hematological disease. Subsequent screen for neurological, dermatological and hemato-oncological involvement revealed no abnormalities. Mediastinal lymph node biopsy and bronchioalveolar lavage (BAL) were both normal. Cardiology review returned no ECG abnormalities and transthoracic echocardiography was normal. Cardiac magnetic resonance (CMR) was performed on a 3-T scanner (Magnetom Skyra; Siemens Healthineers), using Goethe CVI Approaches [5].

**Fig. 1:**
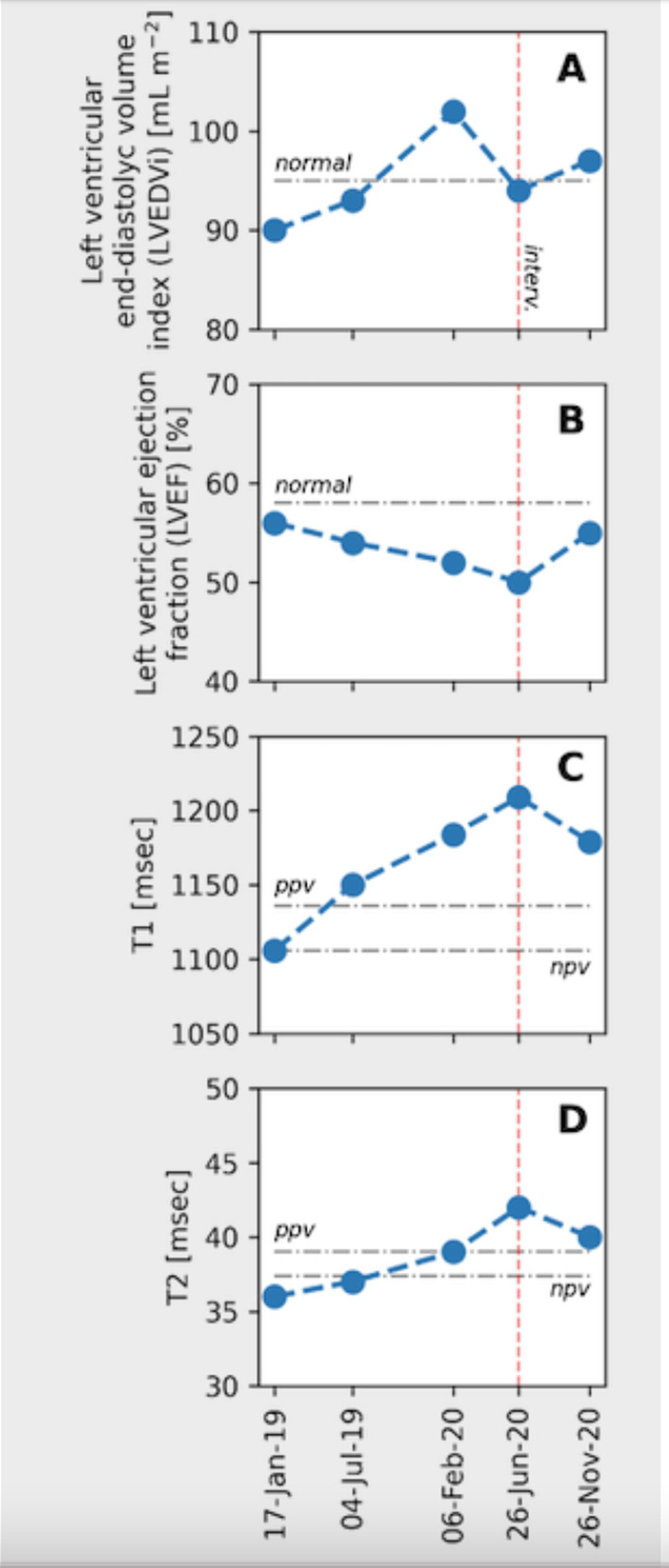
Dynamics of the left ventricular volume, function and cardiac fibrosis, inflammation markers. The horizontal axis shows cardiac MRI scan timepoints, the red vertical line indicating sarcoidosis diagnosis and initiation of the treatment. **A** Left Ventricular End Diastolic Volume Index increased with time, and normalized after anti-remodeling, anti-inflammatory treatment. **B** Left ventricular ejection fraction was decreasing and sub-normalized after treatment. **C** Native T1 value increased drastically and sub-normalized after treatment. D. Native T2 value increased and normalized after treatment. *Ppv-positive predictive value, npv-negative predictive value*

**Fig. 2:**
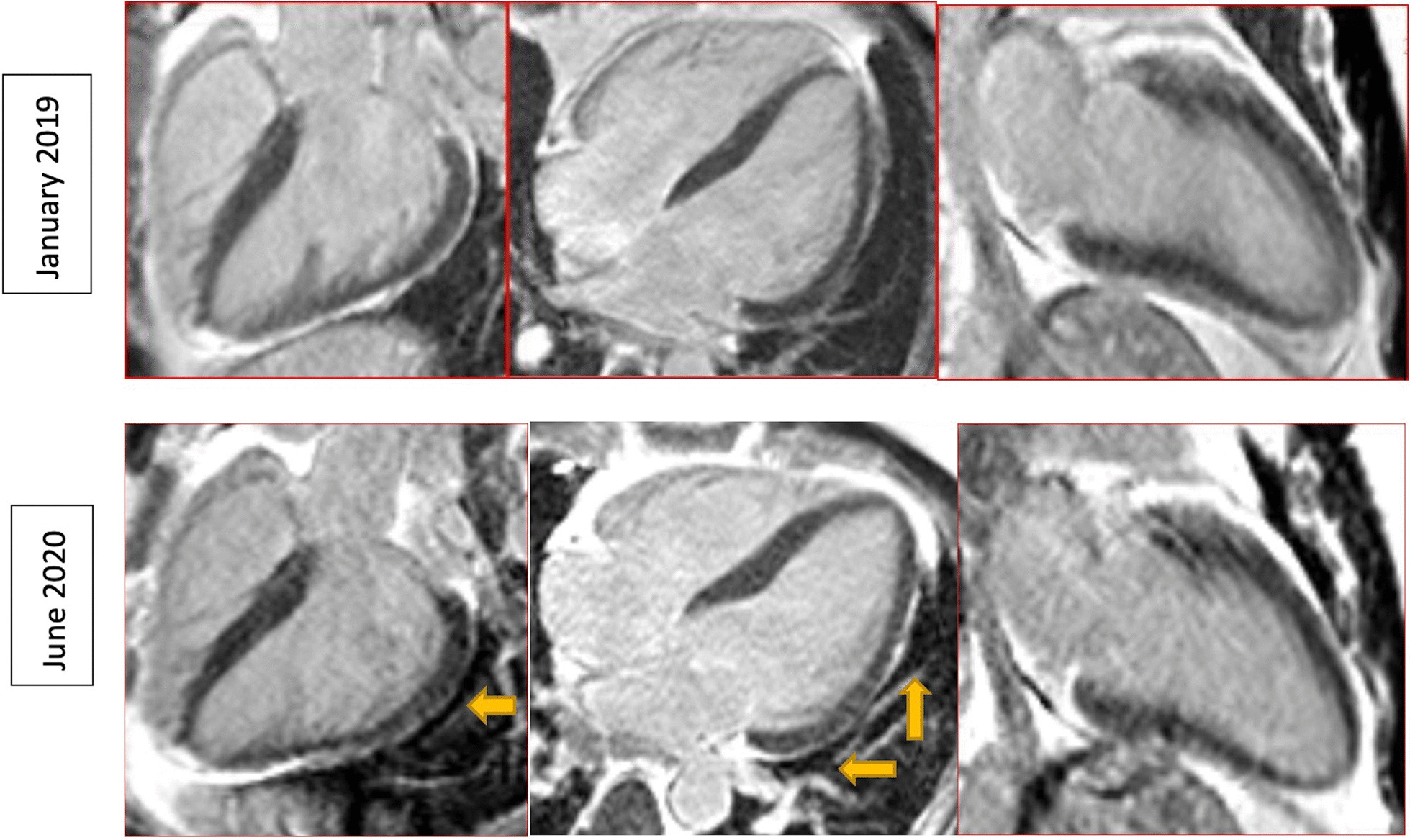
Representative CMR long axis PSIR images. The long axis three-, four- and two- chamber views after injecting gadobutrol, revealing pericardial effusion (orange arrows) and increased LV volume without evidence of non-ischemic scar

**Fig. 3:**
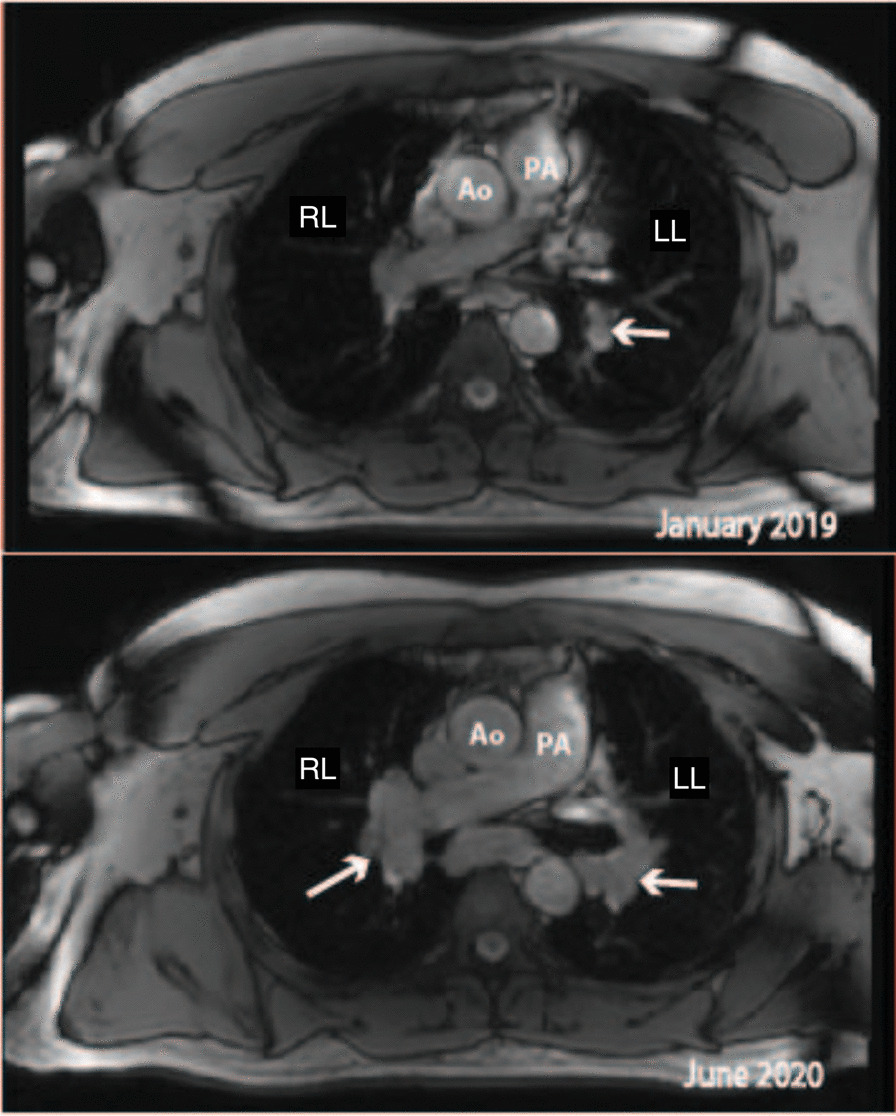
Lymphadenopathy images. CMR localizer in axial plane showed enlarging lymph nodes (white arrows) in the hilar region on both sides over time, suggestive for the diagnosis of sarcoidosis. *Ao- aorta, PA- pulmonary artery, LL-Left lung, RL-right lung*

**Fig. 4:**
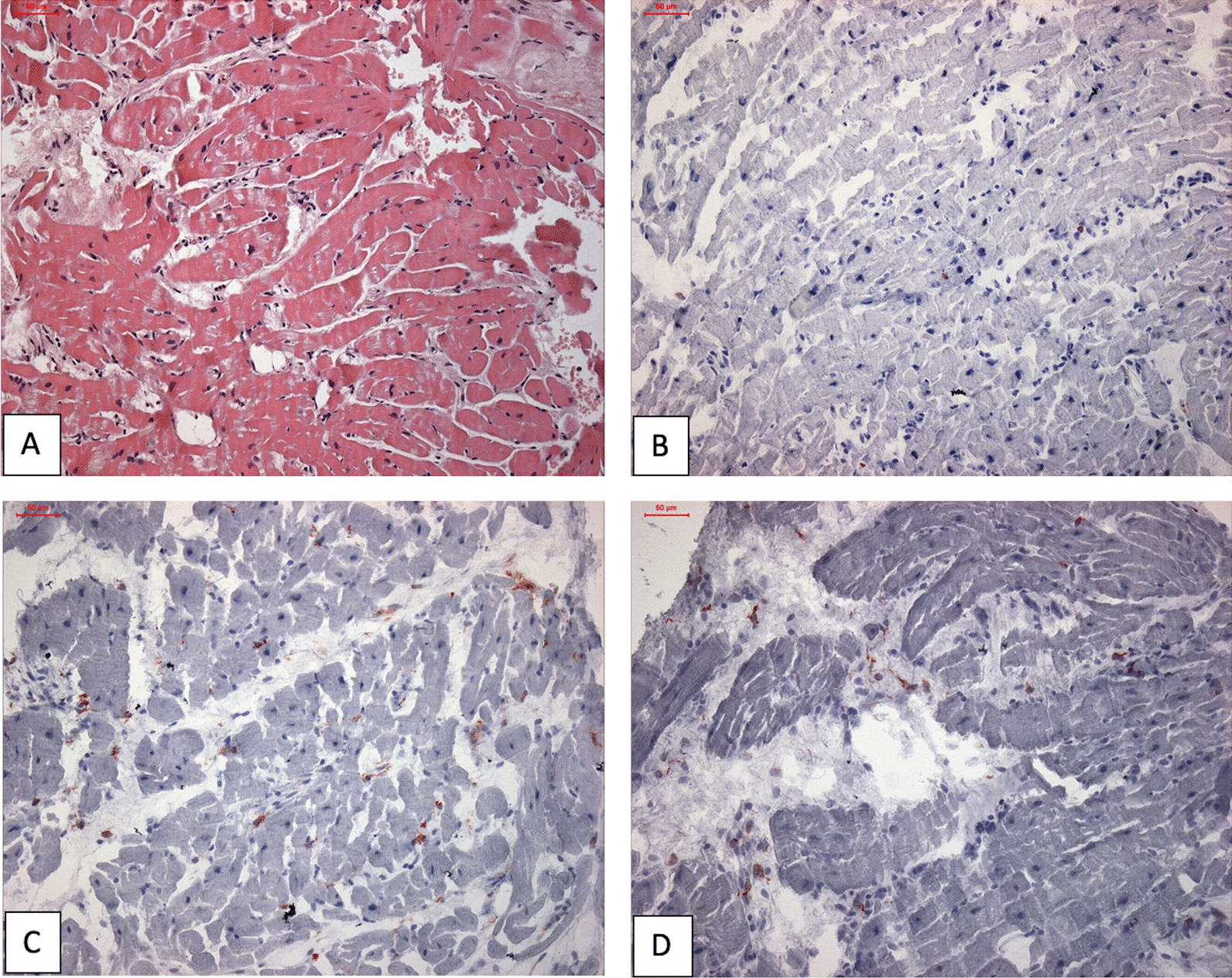
Representative images of (immuno-)histological staining of Endomyocardial Biopsy. **A** Azan staining ×200. The staining shows low fibrosis (blue). **B** HE staining ×200. Staining shows regular cardiomyocytes without evidence of increased inflammatory cells or granulomas. **C** Immunohistological staining CD3-positive T lymphocytes (×200) without an increase of CD3-positive cells. **D** Immunohistological staining of CD45R0-positive t-memory cells (×200) without accumulation of CD45R0-positive cells

Myocardial mapping was acquired in a single midventricular short-axis slice using Goethe CVI modified Look-Locker Imaging (MOLLI) sequence. Initial CMR (CMR 1) (Fig. 1) revealed normal biventricular volumes (age-gender-BSA indexed) and preserved left ventricular ejection fraction (LVEF 56%, normal values: 57–81%) [6]. Right ventricular ejection fraction was normal too (RVEF 50%, normal values: 40–68%). Mildly increased native T1 (1106 ms) and normal T2 (36 ms) indicated diffuse myocardial fibrosis with no active myocardial inflammation (normal ranges: native T1 NPV ≤ 1106 ms; PPV ≥ 1136 ms; native T2 NPV ≤ 37.4 ms; PPV ≥ 39 ms). Late gadolinium enhancement (LGE) imaging (gadobutrol, 0.1 mmol/kg body weight Bayer AG, Leverkusen, Germany) revealed no regional myocardial pathology or scarring. Contrast-free follow-ups revealed progressive drop in LVEF and increase in left ventricular (LV) volumes (Fig. 2), native T1 and T2 values. RVEF remained within the normal range. Due to progressive dyspnea, LVEF decline and worsening of BHL (Fig. 3), together suggesting a high likelihood of systemic sarcoidosis, another round of tissue sampling was initiated, including endomyocardial biopsy (EMB), BAL and mediastinal lymph node biopsy. EMB was performed by sampling 6 tissue samples were harvested from left side of interventricular septum. Representative EMB images are provided in Fig. 4. Immunohistochemically stained sections were evaluated by light microscopy using a LEICA DMRD microscope (Leica; Bensheim, Germany). The image captured via a Sony 3-chip video camera (Sony 3CCD/color-red-green-blue/RGB video camera, Tokyo, Japan) using a Leica C-mount adapter (0.35 magnification) with a Matrox Comet 24-bit color graphics card was processed using the digital imaging analyzing (DIA) program designed on the platform of LUCIA G (V 3.52ab, Nikon, Düsseldorf, Germany). The DIA macros used consisted of three steps: (1) grabbing of image, (2) recognition of artefacts (areas not covered by cardiac tissue) for the calculation of the net myocardial area, and (3) recognition of colored cells. The images for the quantification of infiltrates were grabbed at 200× magnification.

**Fig. 5:**
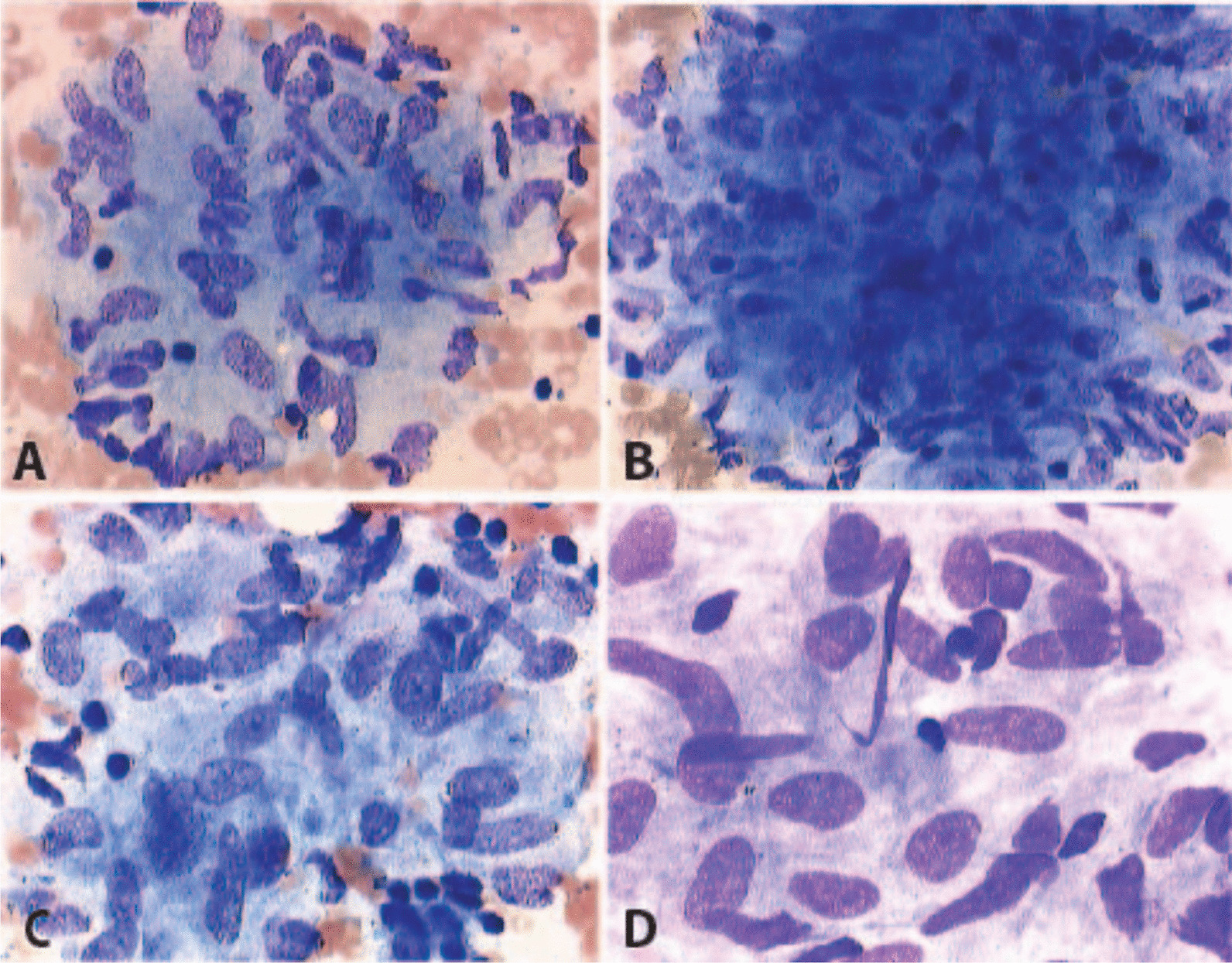
Mediastinal lymph node biopsy- sarcoidosis specific cytochemical result. The images are showing different sizes of activated lymphocytes, epithelioid cells, epithelioid granulomas and eosinophilic granulomas indicating sarcoidosis. (**A**, **B**, **C** 400× magnification, **D** 600× magnification)

Conventional morphology evaluation revealed no pathological changes of cardiomyocyte, including necrosis. The immunohistochemical characterization revealed no lymphocytic inflammatory infiltration (negative staining and counts for CD3, lymphocyte factor A-1, perforin, CD45R0 and MAC-1) or adhesion molecules (HDLA-DR). Azan staining revealed no relevant intra- and perimyocyte fibrosis. Moreover, no sarcoidosis-specific non-caseating granulomas were detected. Qualitative polymerase chain reaction (PCR) test for viral genomes was positive for parvovirus B19-specific DNA sequences, but absence of cDNA indicated no active viral replication. Patient complained hiccups and difficulty in swallowing for several months. Biopsy of mediastinal and hilar lymph nodes was performed using endobronchial ultrasound-guided transbronchial needle aspiration. The evidence of epithelioid and eosinophilic granulomas secured the definite cytopathological diagnosis of systemic sarcoidosis* (*Fig. 5*)*.

## Therapeutic intervention

We initiated cardioprotective treatment with Ramipril 2.5 mg once daily to reduce inflammatory cardiac remodeling in July 2020. Short courses of prednisolone were introduced in December 2020 to control subsequent neurological manifestations (facial palsy).

## Follow-up and outcomes

The result showed rebounding of LVEF and normalized native T1 and native T2 values in the 3 months’ follow-up.

## Discussion and conclusions

Systemic sarcoidosis is a chronic inflammatory condition characterized by histological evidence of non-caseous granulomas. Cardiac involvement with development of heart failure is the leading cause of death in systemic sarcoidosis [1]. Cohort studies have shown that CMR using LGE can reveal non-ischemic patterns of cardiac involvement. LGE often marks an advanced stage of disease which is associated with poor prognosis [7, 8]. Current diagnostic criterion from the Heart Rhythm Society includes non-ischemic myocardial LGE as a diagnostic marker of cardiac involvement [2]. CMR using quantitative mapping helps to inform on the diffuse inflammatory process outside the areas of scar, which can be also detectable in earlier stages of the disease [4]. Informed by serial assessments, we were able to detect disease progression early ahead of scar development. The anti-remodeling effect of Ramipril is largely demonstrated [9]. It explains why we used it in our case before having an accurate diagnosis of that cardiac inflammation with increased LV volumes and progressive drop in LVEF. But we also noticed that proactive cardioprotective treatment could potentially attenuate the myocardial inflammatory reaction with the reduction of parametric mapping values. This anti-inflammatory effect in the myocardium has been explored by some experimental animal studies in the context of post-myocardial infarction or rheumatoid arthritis [10, 11]. Further studies in this field should be considered to determine the role of Ramipril in reducing myocardial inflammation.

The mismatch between clinical manifestations and normal results of EMB is common, as the sensitivity of EMB is very low from 19 to 32% [12]. This low yield is probably due to sampling error: EMB is often performed in the right ventricle, whereas granulomas are mostly located in LV free wall or basal septum [13]. In the present case, however, the EMB was left-ventricular. FDG-PET scanner is another key diagnosis and staging tool for cardiac sarcoidosis, very sensitive for early stages of inflammation [12]. Whereas useful for initial diagnostic work-up, the accessibility, cost and radiation exposure make its use not suitable for follow-up in a young person.

In cardiac sarcoidosis, regional scar formation is a late-stage consequence of myocardial injury. Quantitative CMR helps to detect diffuse fibrosis and inflammation using T1 and T2 mapping. Serial short contrast free examinations may help uncover early disease progression allowing cardioprotective treatment. Future studies are necessary to test the role of imaging markers in guiding therapy and improving cardiovascular outcomes.

## Learning Objectives

1. To understand the potential of quantitative tissue characterization using T1 and T2 mapping to detect early cardiac involvement of systemic sarcoidosis.

2. CMR is a vital tool to noninvasively inform on the diagnosis, progression and treatment response of cardiac sarcoidosis.

## Data Availability

The datasets generated during and/or analysed during the current study are available from the corresponding author on reasonable request.

## Abbreviations

BAL: Bronchioalveolar lavage
BHL: Bilateral hilar lymphadenopathy
CMR: Cardiac magnetic resonance imaging
EMB: Endomyocardial biopsy
FDG-PET: Positron emission tomography with ^18^F-fluorodeoxyglucose
HRS: Heart Rhythm Society
JSSOG: Japanese Society of Sarcoidosis and other Granulomatous Disorders
LGE: Late gadolinium enhancement
LV: Left ventricular
LVEF: Left ventricular ejection fraction
LVEDVi: Left ventricular end-diastolic volume index
NPV: Negative predictive value
PPV: Positive predictive value
